# Primary Closure without Diversion in Management of Operative Blunt Duodenal Trauma in Children

**DOI:** 10.5402/2012/298753

**Published:** 2012-11-05

**Authors:** Katherine Smiley, Tiffany Wright, Sean Skinner, Joseph A. Iocono, John M. Draus

**Affiliations:** ^1^University of Kentucky College of Medicine, Lexington, KY 40536, USA; ^2^Department of Pediatrics, University of Kentucky, Lexington, KY 40536-0298, USA; ^3^Kentucky Children's Hospital and Chandler Medical Center, University of Kentucky, Lexington, KY 40536-0298, USA; ^4^Division of Pediatric Surgery, Department of Surgery, University of Kentucky, Lexington, KY 40536-0298, USA

## Abstract

*Background*. Operative blunt duodenal trauma is rare in pediatric patients. Management is controversial with some recommending pyloric exclusion for complex cases. We hypothesized that primary closure without diversion may be safe even in complex (Grade II-III) injuries. *Methods*. A retrospective review of the American College of Surgeons' Trauma Center database for the years 2003–2011 was performed to identify operative blunt duodenal trauma at our Level 1 Pediatric Trauma Center. Inclusion criteria included ages <14 years and duodenal injury requiring operative intervention. Duodenal hematomas not requiring intervention and other small bowel injuries were excluded. *Results*. A total of 3,283 hospital records were reviewed. Forty patients with operative hollow viscous injuries and seven with operative duodenal injuries were identified. The mean Injury Severity Score was 10.4, with injuries ranging from Grades I–IV and involving all duodenal segments. All injuries were closed primarily with drain placement and assessed for leakage via fluoroscopy between postoperative days 4 and 6. The average length of stay was 11 days; average time to full feeds was 7 days. No complications were encountered. *Conclusion*. Blunt abdominal trauma is an uncommon mechanism of pediatric duodenal injuries. Primary repair with drain placement is safe even in more complex injuries.

## 1. Introduction

 Blunt duodenal trauma remains a relatively rare diagnosis among the pediatric population, accounting for 3 to 5% of all abdominal injuries [1]. Many cases are the result of lap belt or bicycle handlebar injuries, although higher mortality rates have been reported with blunt duodenal injury secondary to nonaccidental trauma [2]. In contrast to adult duodenal traumas, of which greater than 70% are penetrating, the majority of pediatric duodenal injuries are secondary to blunt trauma [3]. Cerise and Scully emphasized that trends in duodenal trauma have demonstrated a gradual increase in severity as a greater proportion are now secondary to motor vehicle accidents, with relatively fewer children experiencing low-velocity handlebar trauma [4]. Their collected cases allowed them to describe three general mechanisms of injury to the small bowel, which include crushing the bowel between the spine and a blunt object, tangential shearing against a relatively immobile segment of bowel, and increased intraluminal pressure causing rupture of a closed bowel loop [4].

 Appropriate treatment of such injuries is hampered both by potential delay in diagnosis and by the controversial nature of optimal surgical management. Often, diagnostic delay is secondary to poor communication between the examiner and the pediatric patient, a lack of specific physical findings suggesting an intra-abdominal lesion, or else the lesion is overshadowed by multiple traumatic injuries. A case series by Kakos et al. [5] reported abdominal pain as the only consistent symptom of blunt small bowel trauma, occurring in twenty-five of twenty-six patients. In cases of blunt duodenal trauma, this lack of specific symptomatology is compounded by the anatomy of the duodenum itself, whose predominantly retroperitoneal location may hide an injury that would be obvious otherwise [3, 6]. The patient may promptly exhibit right upper quadrant pain, progressive tachycardia, and vomiting, but peritoneal signs are often delayed several hours as duodenal contents slowly seep into the peritoneal cavity. the protected anatomic location of the duodenum implies that significant blunt force is needed to produce a small bowel injury, thus raising the likelihood that other abdominal structures, including solid organs, will be injured in the process [7, 8]. One final consideration is the systems-based limitation of referral centers in awaiting the initial workup and transport of patients from less well-equipped facilities, which may contribute to operative delay.

Highlighting the characteristics of blunt duodenal trauma underscores both the importance and difficulty associated with detecting it in a timely fashion. Physicians must have a high index of clinical suspicion, as a delay in operative treatment has been shown to increase morbidity and mortality. One series published by Lucas and Ledgerwooddemonstrated no apparent effects on outcome in patients treated within 24 hours of injury; however, 4 of 10 patients who received operative treatment after 24 hours died, and 3 additional patients within this cohort developed fistulas requiring extended hospitalization [8]. Several imaging modalities have been demonstrated to be helpful in diagnosing blunt duodenal injury, including abdominal plain films, ultrasound, and computed tomography (CT) imaging with oral and intravenous contrast. Classic signs of duodenal injury on plain film include retroperitoneal air outlining the lateral duodenum and right kidney, a partially obscured upper portion of the right psoas muscle, and lumbar spinal scoliosis to the left. However, CT with contrast has more recently become a widely used modality in diagnosing and assessing the severity of blunt duodenal injuries. Luminal contrast materials such as meglumine diatrizoate (Gastrografin, Bristol-Myers Squibb) or barium sulfate may prove helpful as adjuncts in locating a duodenal perforation precisely [1]. Shilyansky et al. [6] demonstrated the ability of contrast-enhanced CT to differentiate duodenal hematoma from perforation, with 9 of 9 patients demonstrating retroperitoneal air or contrast extravasation on CT later being found to have perforation on laparotomy. Likewise, 0 of 10 patients with hematomas demonstrated these signs on CT imaging. 

Once blunt duodenal trauma has been diagnosed, it is the surgeon's task to decide which of a growing number of techniques to employ in its repair. Various methods of staging have historically been proposed to assist with this decision, including the duodenal Injury Severity Scale proposed by Moore et al. [9], (Table 1). 

## 2. Methods 

 This study was performed with Institutional Review Board approval under protocol 120220P1H. A retrospective review of the American College of Surgeons' Trauma Center database was performed to identify all patients with operative blunt duodenal trauma at our institution between 2003 and 2011. Inclusion criteria included age >14 years and duodenal injury requiring operative intervention. Duodenal hematomas that did not require intervention and other small bowel injuries were excluded. Hospital records for those patients requiring operative intervention were collected and reviewed, with data collected on patient demographics, severity and location of injury, mechanism of injury, length to full feeds, length of hospital stay, and complications related to operative intervention. Data are expressed based on the cohort's mean with regard to each parameter measured. 

## 3. Results

**Figure 1:**
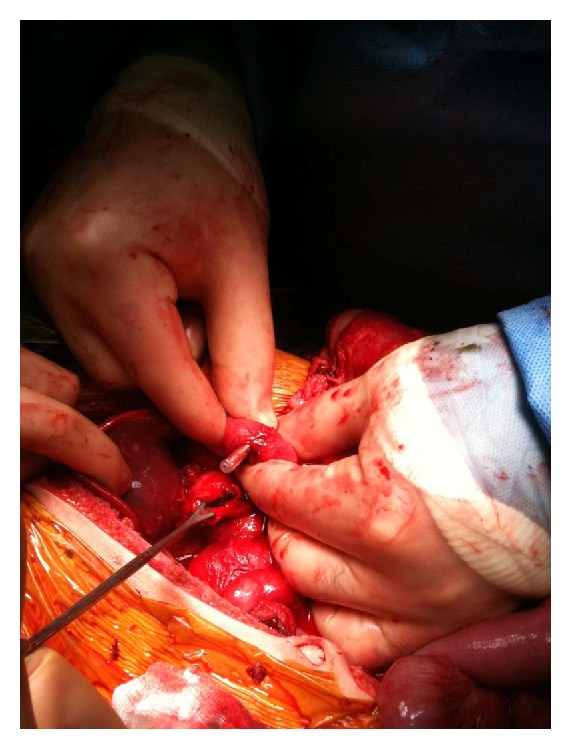
Five-year-old female with complete transection of D1. Orogastric tube is seen exiting proximal end and vein retractor is holding open distal end of injury.

Seven children were identified, through our search of the database, to have undergone operative repair for blunt duodenal injuries within the time specified. The patients were all between 3 and 10 years of age, with a mean age of 6.4 years. Anatomical locations of injury included one patient with a D1 segment injury (Figure 1), three patients with D2 injuries, one with a D3 injury, and two additional patients with greater than one duodenal segment injured. The mechanisms and classifications of each injury, including Injury Severity Score (ISS), as well as the time to full feeds for each patient, are listed in Table 2. All patients underwent primary closure with drain placement, and all had imaging on postoperative day 4–6 with fluoroscopic study of the primary closure to evaluate for leakage of intraluminal contents. None of the patients required reoperation, and no complications were encountered. The average length of hospital stay for the series was 11 days.

## 4. Discussion

Historically, duodenal injuries were often treated aggressively with such technically complex procedures as duodenal diverticulization, which was first described by Donovan and Hagen [10] in 1966 for higher-grade lesions. More modern trends in operative repair demonstrated value in simpler options, including pyloric exclusion and gastrojejunostomy, or primary repair with or without tube duodenostomy [1]. The current literature demonstrates a relative consensus that Grade I injuries such as duodenal hematomas and serosal tears may be treated without operative intervention unless more severe comorbidities or sequelae warrant laparotomy [1, 6]. Higher grade injuries are increasingly being treated with primary closure, also known as duodenorrhaphy, rather than more complex methods of diversion, although some controversy over methodology remains. 

After a decade-long review of blunt duodenal traumas at their institution, Ladd et al. [11] reported an average hospital stay of 24 days for children who underwent primary closure versus 19 days for children with pyloric exclusion, a statistically insignificant difference. Based on their findings, the authors recommended employing pyloric exclusion only when a diagnostic delay of more than 24 hours or injuries greater than Grade III are present. Other authors have reported similar success with primary closure and thus advocate its use for most cases of duodenal injury, even in some large duodenal defects [6, 12]. Carrillo et al. [1] report that Roux-en-Y duodenojejunostomy provides an acceptable alternative for Grade III lesions comprising 50 to 75% of the duodenal circumference, creating a mucosa-to-mucosa anastomosis that functions especially well in repairing large defects in the D2 segment. The most dramatic injuries may warrant resection and anastomosis via duodenoduodenostomy or duodenojejunostomy, occasionally requiring a pancreatoduodenectomy for Grade IV-V injuries involving the distal biliary tree or main pancreatic duct. Even more involved procedures exist as “damage control,” whereby an abbreviated laparotomy is followed with a planned reoperation once the patient is medically optimized [1]. However, the evidence increasingly suggests that these difficult procedures may be reserved for more severe cases, serving as the exception rather than the rule. 

 Blunt abdominal trauma is an uncommon mechanism of pediatric duodenal injuries, often caused by lap belt or handlebar trauma. Based on this limited series at our Level I Trauma Center, attempts at primary repair with drain placement were demonstrated to be safe, even in more complex (Grade II-III) duodenal injuries. By applying the basic principles of successful primary closure used elsewhere in the gastrointestinal tract—namely, clean edges, avoidance of ischemia, and early operation, we were able to employ this simplified technique without significant morbidity and with no mortality. We believe that primary repair is feasible even in more complex cases (i.e., complete transections of the duodenum). In conclusion, comparison of our experience with that of other pediatric trauma centers may help to further elucidate the role of primary closure in repairing blunt duodenal trauma. 

## Figures and Tables

**Table 1:** Duodenal injury severity scale.

Grade	Injury
(I)	Hematoma involving single portion of wall Laceration: partial thickness, no perforation

(II)	Hematoma involving more than 1 portion Laceration: <50% circumference distribution

(III)	Laceration: disruption 50%–75% circumference of 2nd portion Disruption 50%–100% circumference of 1st, 3rd, or 4th portions

(IV)	Laceration: disruption of >75% circumference of 2nd portion Involvement of ampulla or distal common bile duct

(V)	Laceration: massive disruption of duodenopancreatic complex Duodenal devascularization

**Table 2:** Patient demographic data.

Patient data series						
Age	Sex	Mechanism	ISS	Location	Injury	Time to full feeds
7 yo	F	MVC	9	D2	>50% Serosal tear	6 days
5 yo	F	Bicycle	16	D2/D3	Perforation	6 days
7 yo	M	MVC	9	D3	>50% Serosal tear	9 days
8 yo	M	ATV	9	D2	>50% Serosal tear	3 days
10 yo	F	MVC	9	D3/4	>50% Serosal tear	8 days
3 yo	M	MVC	9	D2	Perforation	8 days
5 yo	F	MVC	16	D1	Complete transection	9 days

MVC: motor vehicle collision; ISS: Injury Severity Score; D1–D4 = duodenal segments 1–4.
